# Dual‐focal‐spot single‐detector CT: A simulation study to assess cone‐beam artifacts and noise

**DOI:** 10.1002/acm2.70220

**Published:** 2025-09-01

**Authors:** Baojun Li, Xiangyang Tang

**Affiliations:** ^1^ Department of Radiology Boston University School of Medicine Boston Massachusetts USA; ^2^ Department of Radiology Emory University School of Medicine Atlanta Georgia USA

**Keywords:** cardiac, CT, multi‐detector CT, multi‐source CT, organ‐in‐a‐rotation

## Abstract

**Background:**

Ultra‐wide coverage CT (> 128 detector rows) makes it possible to image a heart or brain in a single rotation but are associated with large cone angles, which can severely degrade the image quality.

**Purpose:**

This study evaluate the image quality and artifact levels of a dual‐focal‐spot single‐detector (DFSSD) CT geometry designed to achieve 140 mm z‐axis coverage, through a simulation study.

**Methods:**

The DFSSD CT system employs two x‐ray focal spots spaced 90 mm apart along the z‐axis and a 100 mm CT detector. It provides 140 mm z‐axis coverage at the iso‐center. A research CT simulation package (CatSim) was employed throughout this study. To compare the image quality, three system geometries were simulated: (A) single focal spot with 40 mm detector (VCT40), (B) single focal spot with 140 mm detector (VCT140), and (C) the DFSSD CT system. A simulated helical body phantom (45 cm x 30 cm x 16 cm) containing various bony structures was used to assess cone‐beam artifacts and noise uniformity across the edge, central, and intermediate slices under different acquisition modes: axial half scan, axial full scan, and helical scan at various helical pitch (0.5, 0.75, 1.0). All images were reconstructed using a cone‐beam filtered back‐projection algorithm with 3D cone‐angle‐dependent pixel‐wise weighting.

**Results:**

Under helical scan conditions (pitch 0.5 to 1.0), all geometries demonstrated similar levels of cone‐beam artifacts and noises. Slightly increased artifacts observed on some slices in VCT140 and DFSSD CT, but overall image quality are acceptable. However, under axial half scan condition, the VCT140 geometry exhibited significantly worse artifacts and image uniformity in intermediate and edge slices compared to VCT40, whereas DFSSD CT showed artifact and image uniformity comparable to VCT40.

**Conclusion:**

Simulation results indicate that the DFSSD CT geometry can achieve 140 mm z‐axis coverage while maintaining image quality similar to the VCT40 system.

## 1 INTRODUCTION

Adequate z‐axis coverage in computed tomography (CT) is crucial for cardiac imaging, particularly when aiming to minimize artifacts caused by cardiac motion. In cases wherein the detector cannot completely cover the heart in single rotation, retrospective ECG‐gating is often required.^1^ However, due to variability in patient heart rates and contrast medium dynamics, motion artifacts can still occur.^2^, ^3^

Determining the optimal z‐axis coverage is essential for various cardiac imaging applications. For instance, imaging the left atrium alone requires approximately 80 mm of z‐coverage (i.e., longitudinal coverage).^4^ In contrast, imaging the entire heart typically demands 120–130 mm of coverage.^5^, ^6^ Given the heart's continuous motion, an additional buffer zone along the z‐axis is recommended, bringing the total to approximately 140 mm. As a result, vendors compete to offer the most detector rows in their CT scanners, leading to what is commonly referred to as the “slice wars.”

Various multi‐source CT systems have previously been proposed and studied, with the number of sources ranging from 3 to 32.^7^, ^8^, ^9^, ^10^ Using anthropomorphic phantoms and/or computer simulations, these studies have demonstrated the key advantages of multi‐source configurations over traditional single‐source systems: (1) substantially improved 3‐D frequency sampling (data consistency), resulting in reduced cone‐beam artifacts such as shading and streaks, and (2) enhanced image and noise uniformity. However, implementing a multi‐source configuration with three or more sources presents significant engineering challenges for x‐ray tube design, likely leading to increased costs and decreased reliability. An alternative approach involves moving a single source to different positions,^9^ but this would likely prolong acquisition times, limiting its clinical applications.

To address these challenges, a dual‐source single‐detector CT system (Spotlight Duo, Arineta, Israel) has been introduced recently, featuring only two focal spots along the z‐axis, designed to achieve 140 mm of z‐axis coverage. We conducted a computer simulation study to assess the image quality and effectiveness of this design. The following sections will detail the geometry and present the findings from our simulation study.

## 2 MATERIALS AND METHODS

### 2.1 Dual‐focal‐spot single‐detector (DFSSD) CT

**FIGURE 1:**
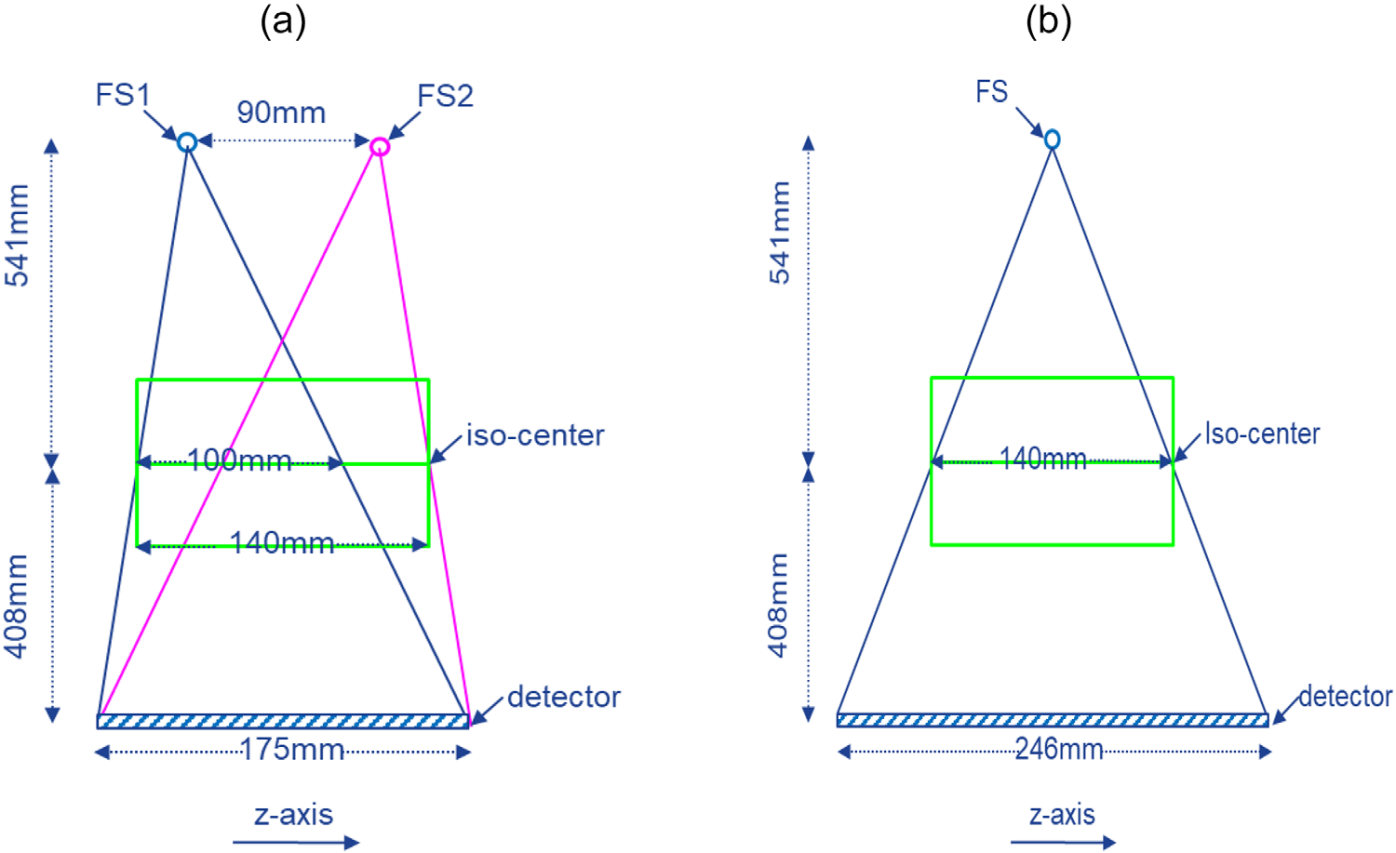
(a) Dual‐focal‐spot single‐detector CT consists of two focal spots spaced at 90 mm apart in z‐axis and a 100 mm detector (iso‐center), which provides 140 mm z‐axis coverage. (b) To provide the same z‐axis coverage, single‐focal‐spot single‐detector CT requires a 140 mm detector (iso‐center) with much larger cone angles.

The DFSSD CT system consists of two focal spots spaced at 90 mm apart in z‐axis and a 100 mm (at iso‐center) third‐generation curved CT detector. As it can be seen in Figure 1a, this geometry can achieve 140 mm z‐axis coverage at the iso‐center, 40% more than the physical dimension of the detector. Compared to the single‐focal‐spot, single‐detector system geometry (Figure 1b), cone angles of the DFSSD CT system are significantly smaller.

### 2.2 Computer simulation

A research CT simulation package, CatSim, was utilized throughout the study.^11^ Table 1 summarizes the parameters used in the computer simulation. Three CT system geometries have been simulated and compared: (a) dual‐focal‐spot single‐detector (DFSSD) (Figure 1), (b) single source with 140 mm detector (VCT140), and (c) single source with 40 mm detector (VCT40). Since the DFSSD CT contains two focal spots, its number of projections doubled that of VCT40 and VCT140. However, the mAs of each projection was reduced by 50% than that of VCT40 and VCT140 in order to maintain the same total x‐ray flux. Other than these differences, all other parameters were kept the same for all three system geometries.

**TABLE 1:** Acquisition parameters simulated in this study.

(a) Parameter	VCT40	VCT140	DFSSD CT
Source‐to‐iso distance (mm)	541	541	541
Iso‐to‐detector distance (mm)	408	408	408
No. of projections	984	984	1968
No. of detector elements in xy‐plane	888	888	888
Dimension of detector element at iso (mm)	0.58 × 0.62	0.58 × 0.62	0.58 × 0.62
kV	120	120	120
Beam spectrum	ANSI standard	ANSI standard	ANSI standard
mAs	240	240	240^a^
Collimation (mm)	40	140	100
Gantry rotation speed (s)	0.35 (axial half scan); 0.5 (helical scans)	0.35 (axial half scan); 0.5 (helical scans)	0.35 (axial half scan); 0.5 (helical scans)
Helical pitch factor	0.5/0.75/1.0	0.5/0.75/1.0	0.5/0.75/1.0
Scan field‐of‐view (cm)	50	50	50
Bowtie	Body (50 cm)	Body (50 cm)	Body (50 cm)
Display field‐of‐view (cm)	50	50	50
Slice thickness	0.625 mm	0.625 mm	0.625 mm
Recon kernel	Standard	Standard	Standard

^a^ 120 mAs per focal spot.

A variety of acquisition conditions were simulated in the study—axial half scan and helical scans with pitch factor ranging from 0.5 to 1.0. These scan conditions covered not only the cases under typical clinical settings but also the worst scenarios.

**FIGURE 2:**
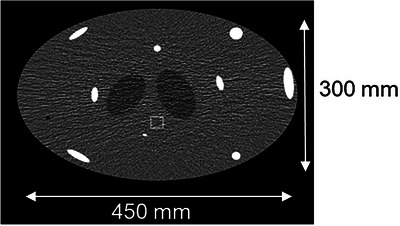
A representative image of the simulated body phantom. Measuring 450 mm x 300 mm x 160 mm, this phantom was quite challenging as it contained many high‐density Teflon rods inserted at varying angles relative to the x–y plane to mimic the 3D orientation of ribs.^12^

A simulated oval‐shaped helical body phantom was utilized in this study (Figure 2). Measuring 450 mm x 300 mm x 160 mm, the phantom consists of a long elliptical cylinder representing the human torso, with strategically placed high‐ and low‐density inserts to simulate anatomical features relevant to artifact analysis. The high‐density Teflon rods near the periphery are inserted at varying angles relative to the x–y plane to mimic the 3D orientation of ribs.^12^ The low‐density cone‐shaped inserts near the center simulate dome of the lungs. These inserts exhibit considerable variation along the z‐axis, thereby enhancing the visibility of cone‐beam artifacts and other reconstruction‐related artifacts. This phantom has been extensively used in prior literature to assess and benchmark CT reconstruction performance, particularly in studies focused on cone‐beam artifact reduction.^13^, ^14^

### 2.3 Image analysis

All images in this study were reconstructed using the cone‐beam FBP algorithm with 3‐D cone‐angle‐dependent pixel‐wise weighting technique to address the data inconsistency problem.^15^, ^16^, ^17^

**FIGURE 3:**
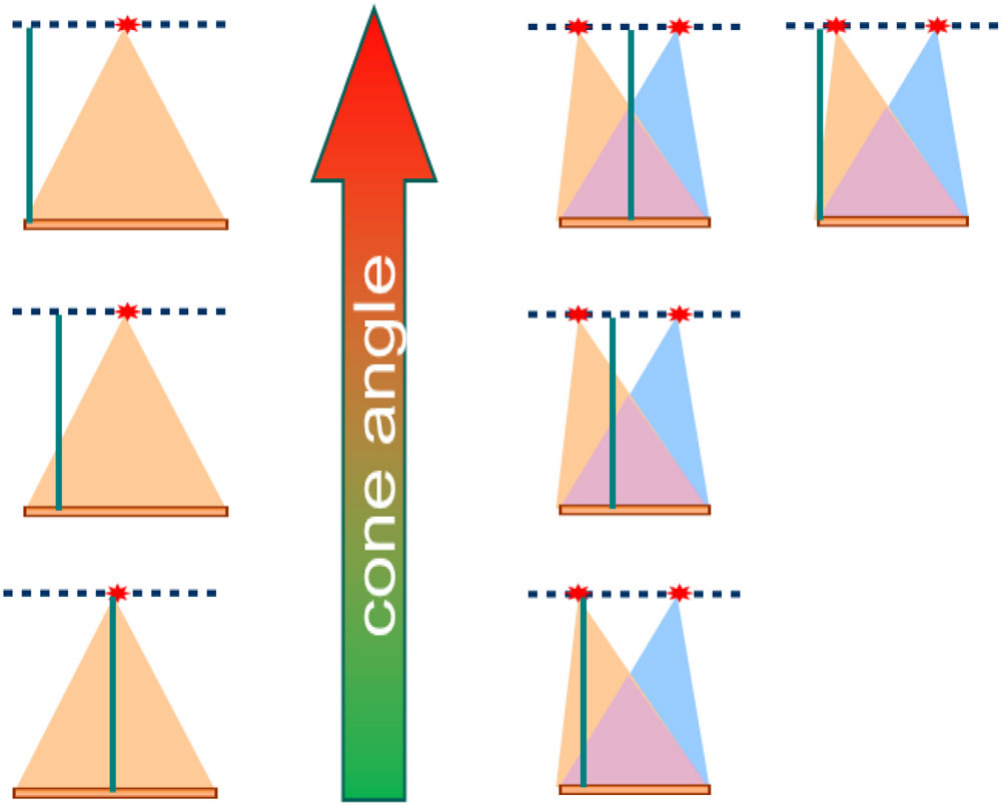
Illustration of different z‐axis slice locations (green vertical lines) with increasing cone angles (pointed by the arrow). (Left column) For geometries employing a single focal‐spot and a single detector such as VCT40 and VCT140, we evaluated the center slice (zero cone angle), the intermediate slice, and the edge slice (maximum cone angle). (Right column) For DFSSD CT, we evaluated the center slice (zero cone angle), the intermediate slice, and the two locations corresponding to the largest cone angles.

**FIGURE 4:**
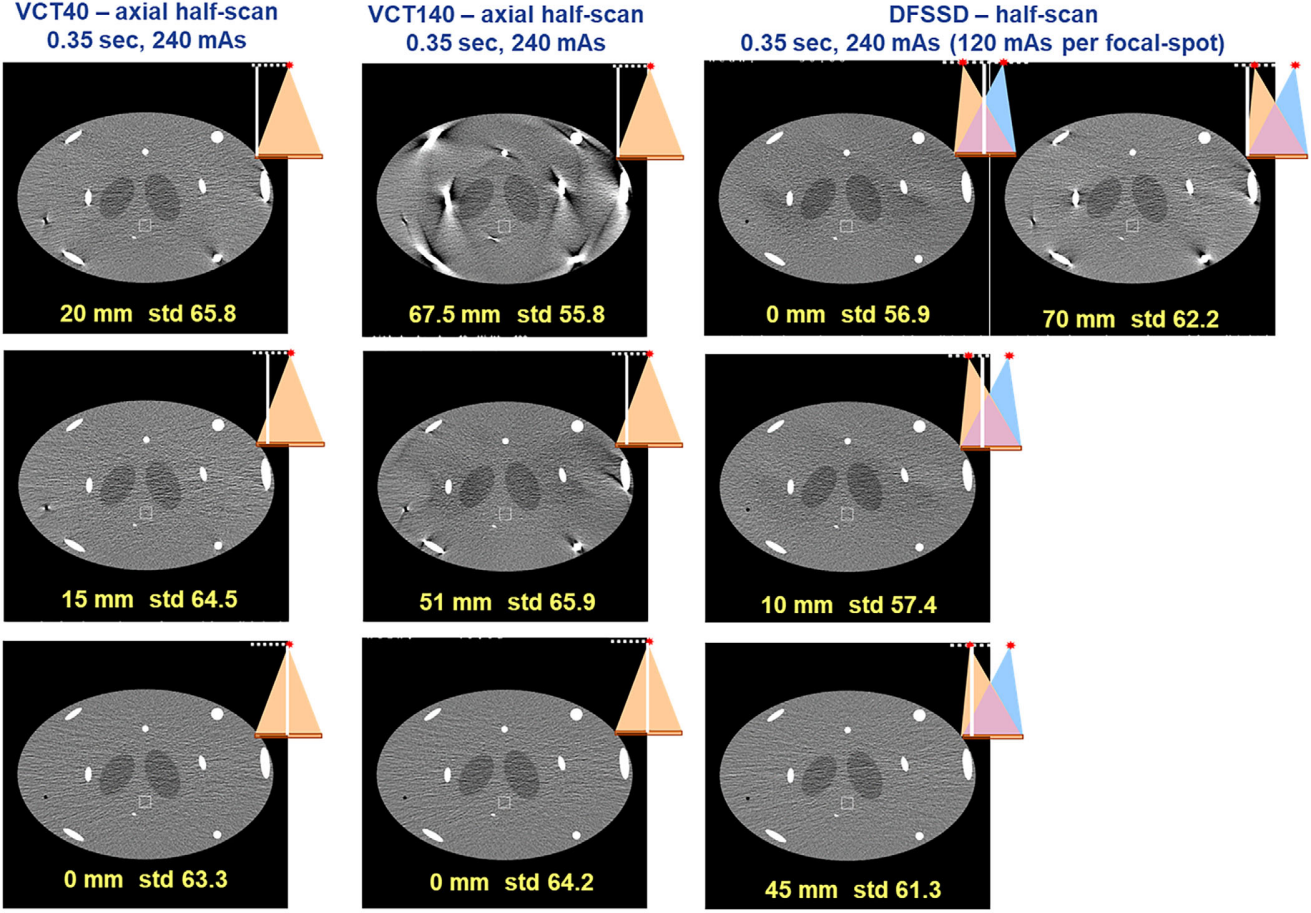
The comparison of axial image quality—axial half scan (window width = 400, window level = 40).

Our evaluation of image quality was centered on cone beam artifacts and noise uniformity. As such, the goal was to assess the image quality at different z‐axis locations ranging from the smallest to the largest cone angle. For geometries employing a single focal‐spot and a single detector such as VCT40 and VCT140, it means we should evaluate the center slice (zero cone angle), the intermediate slice, and the edge slices (maximum cone angle) (Figure 3, Left). For DFSSD CT, since the cone angle increases towards both the detector center and the edge, we evaluated both locations (Figure 4, Right).

## 3 RESULTS

### 3.1 Axial half scan

**FIGURE 5:**
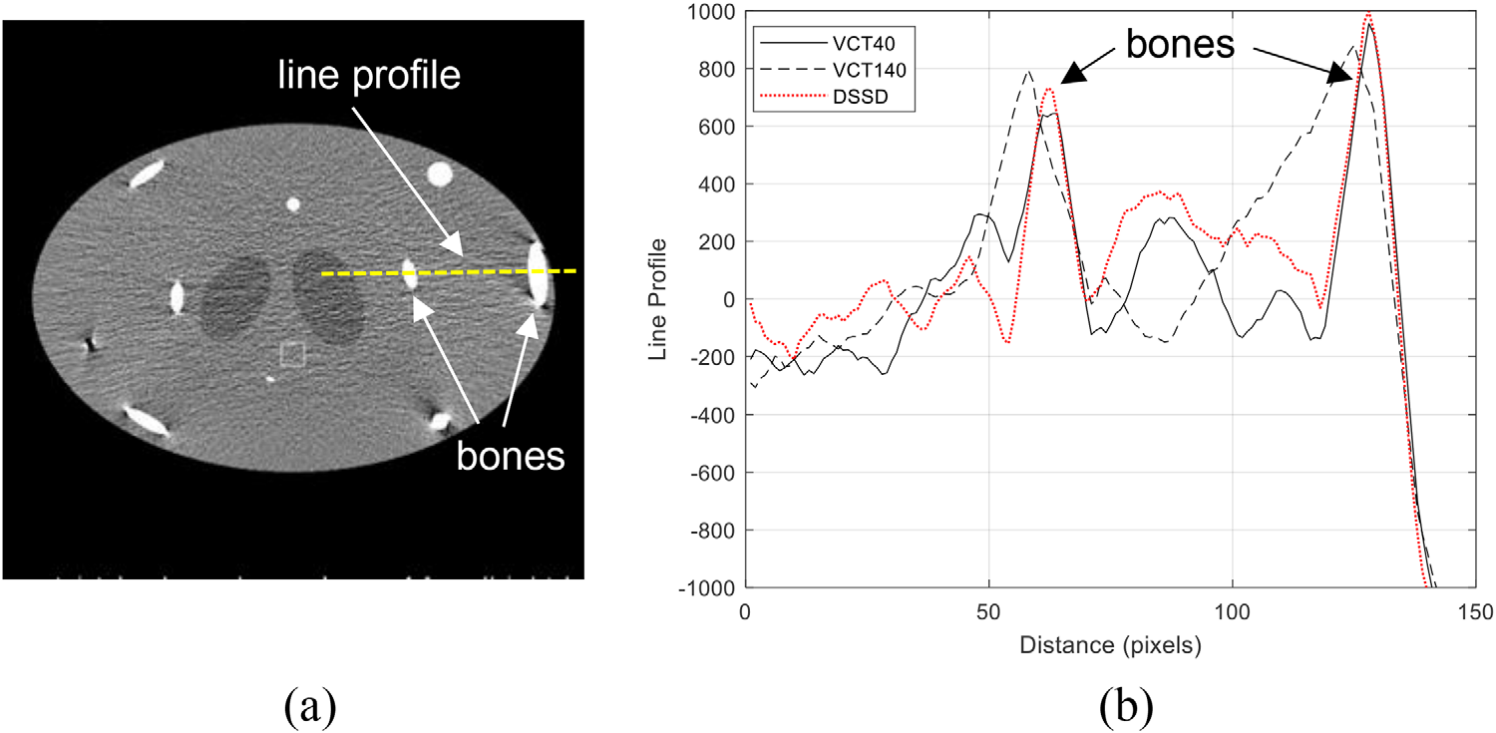
Horizontal line profiles indicated by the dotted yellow line in (a) are displayed in (b) to illustrate the magnitudes of artifacts and image non‐uniformity on the edge slices under axial half scan condition.

Figure 4 shows reconstructed axial images of the simulated body phantom from axial half scans. The left, middle, and right column represents VCT40, VCT140, and DFSSD CT, respectively. The top, middle, and bottom rows are corresponding to the edge slices (maximum cone angle), intermediate slice (intermediate cone angle), and central slices (zero cone angle), respectively. The horizontal line profiles indicated by the dotted yellow line in Figure 5a are displayed in Figure 5b to illustrate the levels of artifacts and image non‐uniformity on the edge slices. The profile plots in (b) were smoothed by a moving average window 5 voxels wide to show the trends in variation more clearly.

It can clearly be seen from Figures 4 and 5 that VCT140 suffers severe cone beam artifacts and image non‐uniformity under axial half scan condition, particularly near the simulated bony structures. Even the intermediate slice demonstrated much worse artifacts than that of VCT40. On the contrary, the DFSSD CT demonstrated comparable cone beam artifacts and image uniformity to VCT40 at various slice locations.

It should be pointed out that a slight noise variation was observed at the edge and intermediate slices of DFSSD CT, owing to the fact that the two beams overlap in a way that creates variation in image flux (Table 2). The maximum percentage noise deviation from that of VCT40 were 13.4%.

**TABLE 2:** Comparison of image noise levels. Values in parentheses indicate the percentage deviation from those of VCT40.

(b)	VCT40	VCT140	DFSSD CT
Axial half scan			
Edge slice	65.8	55.8 (−15.2%)	56.9 (−13.4%)/62.2 (‒5.5%)
Intermediate slice	64.5	65.9 (2.2%)	57.4 (−11%)
Center slice	63.3	64.2 (1.4%)	61.3 (3.1%)
Helical pitch 0.5	37.5	36.2 (−3.5%)	41.1 (9.6%)
Helical pitch 0.75	37.2	35.7 (−4%)	40.2 (8%)
Helical pitch 1.0	52.8	52.7 (−0.2%)	50.3 (−4.7%)

### 3.2 Helical scan

**FIGURE 6:**
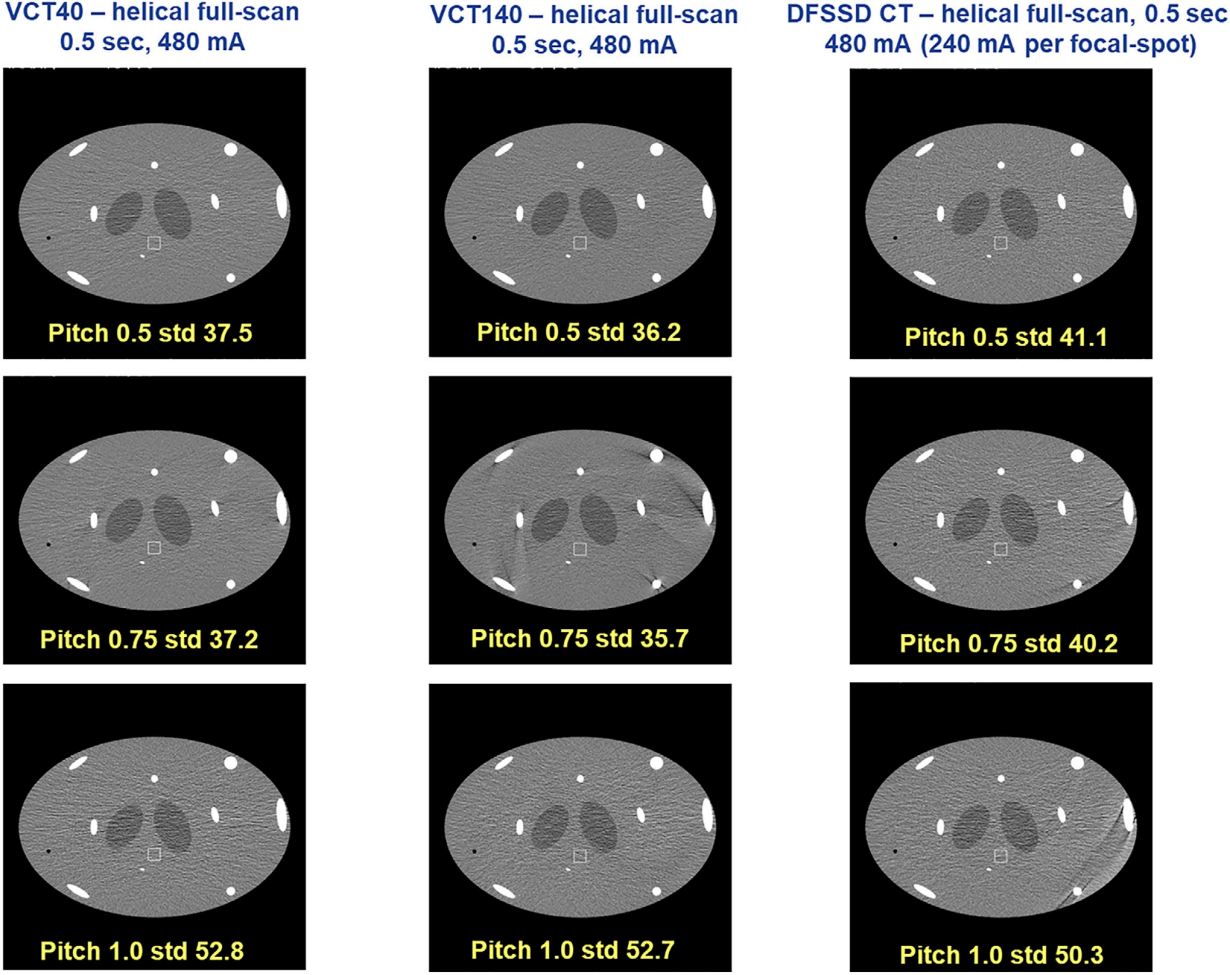
The comparison of helical image quality (window width = 400, window level = 40). (Top row) pitch = 0.5. (Middle row) pitch = 0.75. (Bottom row) pitch = 1.0.

Figure 6 displays the comparison of helical image quality. The top, middle, and bottom row represents helical pitch 0.5, 0.75, and 1.0, respectively. Under various helical scan conditions, all three systems produced similar image quality, as evidenced by the negligible artifact levels and comparable noise levels, indicating that an increase in the helical pitch did not negate the image quality.

## 4 DISCUSSION

In this study, we evaluated a dual‐focal‐spot single‐detector CT geometry designed to achieve 140 mm z‐axis coverage. Our simulation results indicate that, under various pitch values (0.5/0.75/1.0), the DFSSD geometry exhibits noise uniformity and artifact levels comparable to the VCT40 system. Notably, even under the challenging axial half‐scan condition, the DFSSD CT maintains similar artifact levels and acceptable noise uniformity compared to VCT40. In contrast, the VCT140 geometry, while achieving the same 140 mm z‐axis coverage, shows significantly increased cone‐beam artifacts and worse noise uniformity, particularly in the intermediate and edge slices. These findings suggest that the DFSSD CT geometry may offer superior image quality compared to VCT140 for cardiac imaging applications that demand extended z‐axis coverage.

One of the key advantages of DFSSD CT is its potential for cost savings, particularly in relation to the CT detector. The DFSSD CT uses a 100 mm detector to achieve 140 mm z‐axis coverage, representing a 40% reduction in detector‐related costs. Given that the detector accounts for approximately 50% of the total system cost,^18^ this reduction is significant. Although the x‐ray tube in DFSSD CT is more complex and may incur higher costs—possibly doubling compared to standard designs—it typically comprises only around 10% of the total system cost.^18^ Therefore, even with increased x‐ray tube expenses, the overall cost saving with the DFSSD CT geometry remains substantial. More importantly, these financial benefits are coupled with improved image quality, further enhancing the value proposition of this geometry.

Our study has several limitations. First, we did not evaluate the impact of newer reconstruction algorithms, such as model‐based iterative reconstruction, compressed sensing, or deep learning techniques. While we used the same reconstruction algorithm across all systems to ensure a fair comparison, future studies should explore whether advanced algorithms could further enhance the image quality of both DFSSD CT and VCT140. It is worth noting that while we hypothesize that improvements may be constrained by the fundamental physical limitations associated with cone angle, this assumption requires further empirical validation. Second, we did not investigate the effect of the DFSSD CT geometry on spatial resolution. Although we have no immediate reason to suspect that spatial resolution would be significantly degraded by this geometry, this remains an untested aspect of our design. Future research should address this gap to comprehensively evaluate the potential trade‐offs associated with the DFSSD CT.

## 5 CONCLUSION

Our findings suggest that the dual‐focal‐spot single‐detector CT offers a promising alternative for achieving cardiac imaging with improved image quality and significant cost savings compared to other CT geometries. Further studies are needed to explore the integration of advanced reconstruction algorithms and to assess the impact on spatial resolution, ensuring that this geometry meets the rigorous demanding of clinical practice.

## AUTHOR CONTRIBUTIONS

The corresponding author (Baojun Li) contributed substantially to the conception and design of the work, the acquisition of data for the work, the analysis of data for the work, and drafting the work. The co‐author (Xiangyang Tang) made substantial contribution to the conception and design of the work, the analysis of data for the work, and drafting the work.

## CONFLICT OF INTEREST STATEMENT

The authors declare no conflicts of interest.

## ETHICS STATEMENT

Not applicable as this is a simulation study.

## Supporting information



Supporting Information

## Data Availability

A copy of the CatSim configure file used to set up the simulation can be found in the Appendix.
